# The Challenge of Small-Scale Repeats for Indel Discovery

**DOI:** 10.3389/fbioe.2015.00008

**Published:** 2015-01-26

**Authors:** Giuseppe Narzisi, Michael C. Schatz

**Affiliations:** ^1^New York Genome Center, New York, NY, USA; ^2^Cold Spring Harbor Laboratory, Simons Center for Quantitative Biology, Cold Spring Harbor, New York, NY, USA

**Keywords:** next-generation sequencing, sequence assembly, sequence analysis, variant detection, indel mutation, repetitive sequences, nucleic acid

## Abstract

Repetitive sequences are abundant in the human genome. Different classes of repetitive DNA sequences, including simple repeats, tandem repeats, segmental duplications, interspersed repeats, and other elements, collectively span more than 50% of the genome. Because repeat sequences occur in the genome at different scales they can cause various types of sequence analysis errors, including in alignment, *de novo* assembly, and annotation, among others. This mini-review highlights the challenges introduced by small-scale repeat sequences, especially near-identical tandem or closely located repeats and short tandem repeats, for discovering DNA insertion and deletion (indel) mutations from next-generation sequencing data. We also discuss the de Bruijn graph sequence assembly paradigm that is emerging as the most popular and promising approach for detecting indels. The human exome is taken as an example and highlights how these repetitive elements can obscure or introduce errors while detecting these types of mutations.

## Introduction

Enormous advances made over the last decade in next-generation sequencing technologies and computational variation analysis have made it feasible to study human genetics in unprecedented detail. These technologies have enabled the sequencing of many thousands of human genomes to examine the genetics of healthy and diseased human populations. This has included sequencing thousands of healthy individuals of different ancestries from around the world (The 1000 Genomes Project Consortium, 2010; Khurana et al., 2013), along with detailed studies of cancer^1^, autism (Iossifov et al., 2014), and schizophrenia (Schizophrenia Working Group of the Psychiatric Genomics Consortium, 2014), among many other projects. While historically genomic studies have focused on single nucleotide polymorphisms (SNPs) due to their prevalence and relative technical simplicity, a recent trend has been to study the role of insertion and deletion (indel) mutations. Already these projects have discovered indels to be ubiquitous in genomes, occurring nearly as frequently as SNPs, but with great diversity in size ranging from single base indels through larger events covering much larger regions (Montgomery et al., 2013). Indel mutations are especially important because they have been implicated in dozens of diseases through small frameshift mutations as well as larger indels that radically alter genes, change splicing and binding sites, or disrupt other important genomic sequences.

Most of the commonly used approaches for finding mutations from next-generation sequence data align one read at a time to the reference genome and then scan the alignments to identify any mutations (DePristo et al., 2011). This analytical framework works well for identifying simple mutations, as reads with a few mutated bases can generally be correctly aligned to a genome across the mutation. However, for indel analysis, this process becomes less and less effective. In the case of a larger insertion reads supporting the mutation will contain fewer and fewer bases matching the reference and therefore increasingly fail to map. A large deletion also leads to mapping complications, because even though the read consists of bases from the reference, there may not be enough bases to unambiguously map to both sides of the deletion forcing the aligner to instead trim or “soft clip” the reads. Consequently, insertions or deletions of more than a few bases are challenging to discover using standard alignment-based methods, and recent approaches have instead focused on assembly techniques to recover them instead.

Repetitive sequences in the genome also significantly complicate both sequencing and analysis accuracy (Treangen and Salzberg, 2011). Repeats of all classes complicate the mapping process as they introduce ambiguity into the true position of a read potentially reducing the sensitivity of our ability to discover indels or other mutations. Repeats, if not analyzed properly, can also introduce false positives by suggesting the presence of an artificial indels between repetitive elements and decrease the specificity of variant calling methods. Simple tandem repeats (STRs) are especially challenging genomic sequences to sequence and analyze, as they have a substantially greater sequencing error rate than other sequences and are prone to polymerase slippage that can artificially extend or contract the length of the repetitive element (Ellegren, 2004). For example, if a locus should consist of 10 adenines, during the sequencing process reads may be generated with just 9 or even 11. Downstream algorithms examining the sequencing data around STRs may misinterpret the sequencing error as an indel polymorphism in the genome if they are not aware of such effects.

Finally, detection of *de novo* and somatic mutations, although conceptually simple tasks, pose additional challenges within small-scale repeats due to technical and algorithmic problems that can easily introduce false-negative variants. For example, strand biases at the sequencing stage can introduce allele imbalance favoring the reference allele over the mutation at a specific locus in the normal sample with the negative effect of introducing false-positive somatic calls in the tumor. Similarly, calling *de novo* mutations within repeat structures, in particular STRs, is complicated by noise introduced by sequencing errors which can mask (by chance) a true *de novo* mutation by generating the same mutation signature in the parent. Consequently, many large-scale studies currently avoid calling *de novo* mutations at those highly variable sites.

## Indel Discovery – The Era of Micro-Assembly

**Figure 1:**
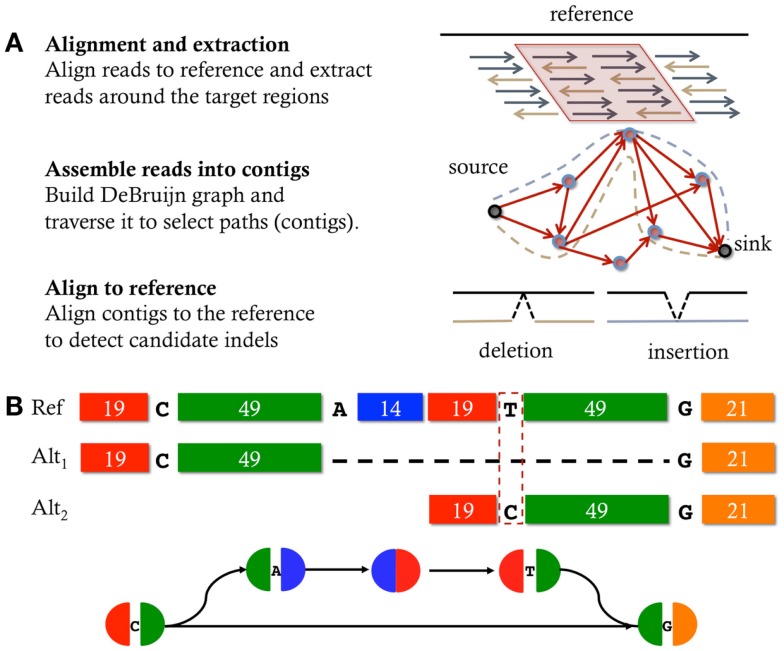
**Micro-assembly**. **(A)** typical workflow of the micro-assembly strategy: given a specific region of interest, the reads, previously aligned to the reference, are extracted, assembled, and then contigs are aligned back to the reference to discover mutations. **(B)** Schematic representation of a bubble in a De Bruijn graph induced by a heterozygous mutation within a repetitive sequence composed of two near-identical copies (with 1 bp mismatch) that are 15 bp apart. Each block represents a sequence; sequences that have the same base pair composition have the same color; the length of each sequence (in base pairs) is reported inside each block. Each node of the graph is colored according to the order and sequence composition contained in it. Simple (non-branching) paths are represented in a single node. Alt1 and Alt2 are two alternative alignments of the sequence contained in the bottom side of the bubble, where the dashed line represents the alignment gap. The top side of the bubble matches the reference. Any *k*-mer longer than the longest identical repeat (>49 bp) and shorter than the longest near-identical repeat (<69 bp) would create a bubble like the one depicted **(B)** where a jump is allowed from the first copy of the near-identical repeat to the second copy.

In an effort to extend the power of detectable mutation using short reads from next-generation sequencing technologies (e.g., Illumina), assembly based variant detection techniques are now becoming a popular solution. The strategy employed by these methods is to perform localized sequence assembly, micro-assembly, of the reads mapping around the location of the candidate mutation. Recently developed tools that use this strategy include Scalpel (Narzisi et al., 2014), GATK HaplotypeCaller^2^, SOAPindel (Li et al., 2012), Platypus (Rimmer et al., 2014), ABRA (Mose et al., 2014), TIGRA (Chen et al., 2014), DISCOVAR (Weisenfeld et al., 2014), and Bubbleparse (Leggett et al., 2013). Figure 1A illustrates the general workflow followed by tools that employ micro-assembly. The first step consists in performing a fast alignment of the reads to the reference genome, typically using BWA (Li and Durbin, 2009); however, these alignments are not directly used to identify mutations but instead the purpose is to localize the analysis by identifying all the reads that have similarity to the given locus. Once a region of interest has been selected, the reads are extracted, including soft-clipped reads and reads that fail to map but are anchored by their mate. The de Bruijn graph of those reads is then constructed by decomposing the reads into overlapping *k*-mers, and then explored to select candidate paths that contain mutations. Finally, the assembled sequences are aligned back to the reference to detect the correct signature for the mutation.

Although all the above-mentioned tools follow this paradigm, they differ in many important aspects. Two key differences are the selection of the *k*-mer size used for assembly and the way repeat sequences are handled. For example, SOAPindel tries to reconnect a broken path in low-coverage regions by searching for unused reads with gradually shorter *k*-mers until a path is formed or the lower bound on *k*-mer length has been reached. Similarly in TIGRA, the user can specify the list of *k*-mer sizes to use; however, this tool has been tailored for breakpoint detection without reporting the indel sequence. GATK HaplotypeCaller by default attempts to build two separate graphs, using *k*-mers of 10 and 25 bases in size; however, other *k*-mer sizes can be specified from the command line. Scalpel instead employs a self-tuning *k*-mer strategy that is coupled with a meticulous repeat composition analysis in order to reduce errors in highly repetitive regions. In contrast to SOAPindel that uses a decreasing size of *k*-mer values, Scalpel starts with a small value (default *k* = 25) and if a repeat structure (either perfect or near-perfect up to a few mismatches) is detected, the graph is discarded and a larger *k*-mer is selected. This process continues until a “repeat-free” graph is constructed or a maximum *k*-mer length is reached, and in the latter case, the region is discarded as undetectable. GATK HaplotypeCaller uses a similar iterative strategy to Scalpel to avoid false-positives indels within repeats by trying a larger *k*-mer when a cycle is detected in the graph. However, only perfect repeats (cycles in the graph) are checked by the GATK HaplotypeCaller, while nearly identical repeats can still mislead the algorithm to generate false-positive calls. ABRA also performs a localized assembly of the reads for genomic regions of size ≤2 kb. Similarly to Scalpel and GATK HaplotypeCaller, all non-cyclic paths through the graph are traversed and, in case a cycle is detected, the region is iteratively reassembled using increasing *k*-mer sizes until the cycle non-longer exists. Platypus integrates the colored de Bruijn graph methods initially developed for Cortex (Iqbal et al., 2012) to also perform a local assembly in small regions (by default 1.5 kb) using a fixed *k*-mer (15 by default). A revised DFS traversal of the graph is used in Platypus to avoid loops and to generate only non-self-intersecting paths. DISCOVAR also involves initial alignment of reads to the genomic regions followed by careful local assembly. Similarly to Platypus, DISCOVAR combines the detection of SNPs and indels into a unified framework. Using a combination of longer reads and improved error-correction algorithms, DISCOVAR demonstrates increased power compared to GATK and Cortex to detect challenging variants located in low-complexity sequences and segmental duplications. However, DISCOVAR is designed to work only with PCR free 250 bp paired-end reads, which are not commonly available. Finally, Bubbleparse, although it is not exactly a micro-assembly method, also attempts to identify SNPs and indels independent of a reference genome using the de Bruijn graph implementation in the Cortex framework, but it does not specifically evaluate the repetitive content surrounding a candidate indel, and was reported to have a high false-positive rate for indels (Leggett et al., 2013).

By assembling longer stretches of DNA sequence around the mutation, micro-assembly techniques allow more accurate alignments and interpretation of the detected mutations and extend the power of detectable indels ≥30 bp. However, like alignment-based methods, these techniques are also susceptible to errors when calling mutation within small-scale repeat sequences, specifically short tandem repeats (STRs) and localized near-identical repeats. For example, a comparative assessment (Narzisi et al., 2014) demonstrated through a large-scale re-sequencing experiment that SOAPindel has a high error rate within repeat structures. In this review, we start by discussing some classes of repeats that can be found in the human exome. We then show examples of the type of errors introduced by these repetitive structures and we provide recommendation on how to reduce or avoid the errors.

## Repetitive Structures in the Human Exome

**Figure 2:**
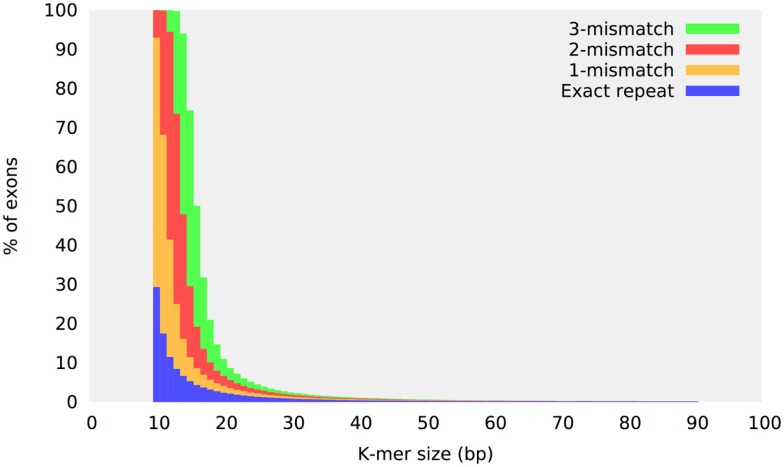
**Repeat content in the human exome**. Repeat content distribution in the human exome target regions as a function of the *k*-mer size. The sequence of each target exon is analyzed to check for the presence of a repeat structure within the same region defining the exon. The *y*-axis reports the percentage of those exons that have been found to contain an identical or near-identical repeat of size *k* (up to three mismatches).

Repeats are the most difficult sequences to assemble and the specificity of any indel detection method is correlated to its ability to detect and analyze repetitive sequences correctly. Although the exome sequence composition is generally assumed to be relatively simple compared to the rest of the human genome, 30% of exons have a perfect 10 bp or larger repeat (Narzisi et al., 2014). More significantly, the number of near-identical repeats (sequences which differ from each other by just a few bases) increases substantially if more mismatches are permitted. Figure 2 shows the percent of locally repetitive human exons as a function of different *k*-mer values and maximum number of mismatches. Each exon target is exhaustively analyzed to check for the presence of an identical or near-identical repeat (up to a maximum of three mismatches) in the same region defining the exon. The *y*-axis reports the percentage of those exons that have been found to contain a repeat of size *k* (*x*-axis). Since the presence of repeats is confined only inside each exon, this analysis demonstrates a substantial level of locally repetitive sequences in the human exome. The two main classes of repeat structures that contribute to this plot are near-identical repeats and STRs. Given the generally low error rate of the Illumina sequencing technology, allowing 3-mismatches for a 10-mer (30% error rate) would seem to not be realistic for a sequencing study. However, this repeat analysis must be examined in the context of performing sequence assembly using de Bruijn graph method, where a perfect match is required for two overlapping *k*-mers.

## Near-Identical Closely Located Repeats

The first major class of repeats that can confound indel discovery techniques is near-identical repetitive sequences that are localized within an exon or other small spans. This type of structures can introduce artifacts in the assembly graph that mislead such methods to make false-positive calls. Figure 1B shows an example of a near-identical repeat that can be misinterpreted as a large deletion. The key observation is that the beginning of this sequence is a nearly identical 69 bp repeat with just 1 bp different between the two copies that are 15 bp apart. The sequence is segmented as 19-C-49-A-14-19-T-49-G-21 where 19 and 49 are 19 and 49 bp identical repeats, separated by a 15 bp unique sequence (A, C, T, G are the typical bases). Since the longest exact repeat is 49 bp long, one would expect that using *k*-mer = 55 should be large enough to correctly assemble reads sampled from this sequence. However, if the sequencing data also contains reads with sequence 19-C-49-G because of a single base A to G change from the expected 19-C-49-A from sequencing error or true mutation, it can be wrongly interpreted as an 84 bp deletion of the A-14-19-T-49 internal segment.

The reason for this ambiguity and other false positives is that the de Bruijn graph is constructed using perfect matches of length *k* *−* 1. So any *k*-mer longer than the longest identical repeat (>49 bp) and shorter than the longest near-identical repeat (<69 bp) would create a bubble like the one depicted Figure 1B where a jump is allowed from the first copy of the near-identical repeat to the second copy. When aligned end-to-end to the reference, the sequence associated to the branch can be aligned in two different ways, one showing a large (false-positive) 84 bp deletion and the other one showing a single nucleotide variation. To reduce the chance to make false-positive indel calls in these regions, it is essential to evaluate the presence of near-identical repeats in either the reference or the assembled sequences. Then, if this type of ambiguity is detected, a decision must be taken to discard the region, flag the candidate mutations as having low quality, or use a *k*-mer size (if any) that avoids the creation of the false-bubble due to the near-identical repeat (e.g., Scalpel).

## Short Tandem Repeats

Short tandem repeats, also known as microsatellites, are highly mutable genetic elements that consist of multiple repeating copies of elements composed of 1–6 nucleotides. Indels that alter the length of the repeat motif have been linked to more than 40 neurological diseases (Pearson et al., 2005). Despite recent advances in sequencing technology, STR variation pose remarkable challenges to variant detection methods compared to other classes of mutations, such as single nucleotide and copy number variation. Discovery of genetic STR variation with short-read sequence data is confounded by (1) the difficulty of uniquely mapping short, low-complexity reads, (2) the high rate of STR amplification error (e.g., homopolymers) due to replication slippage events and which result in high variability in the number of repeat elements (Mirkin, 2007), and (3) the fact that the spontaneous mutation rate of STRs can reach 1/500 mutations per locus per generation (Ballantyne et al., 2010). Due to these effects, distinguishing between sequencing errors and true mutations within STRs is the major challenge faced by indel detection methods. Moreover, even after a candidate locus for an STR mutation has been identified, the associated indel haplotype description can still have an ambiguous position. For example, if there is a 1 bp deletion in a long homopolymer (…AAAAAA…), deleting any A will give rise to the same haplotype but just with a different position. A more complex example which gives rise to two logically equivalent 3 bp deletions is

$$\begin{array}{ccc} \text{ref: } & & \text{AAACTGGAGGTTGC}\\ {\text{alt}}_{1}\text{: } & & \text{AAACT– – –GGTTGC}\\ {\text{alt}}_{2}\text{: } & & \text{AAACTGG– – –TTGC} \end{array}$$

Note that two different 3 bp sequences can be deleted (GGA or AGG) at two different locations generating the same alternative sequence. Since different methods might report different signatures for the same indel, these examples show how essential is to normalize the signature (typically left-normalization) when comparing indels.

Relatively few computational tools have been developed to specifically deal with the complexity of calling in STR regions. RepeatSeq (Highnam et al., 2013) and lobSTR (Gymrek et al., 2012) are the two most recent ones. In order to reduce the error rate at STR loci both methods use statistical modeling to empirically derive the sequencing error model. Comparisons with standard aligners such as BWA, Bowtie, and Novoalign, demonstrate that these aligners are biased toward the detection of the reference allele and specialized tools are required. Moreover most of the highly polymorphic STRs have length of 20 bp or longer, and unfortunately these sizes are the most prone to polymerase slippage and alignment artifacts (Gymrek et al., 2012). The major obstacle for STR profiling is the limited read length of current widely used sequencing technologies. A read must span the complete STR sequence in order to be detected with alignment. Micro-assembly is a promising approach to extend even further the spectrum of detectable STRs mutations and we expect micro-assembly tools specialized for STRs profiling to be developed in the near future.

## Conclusion

The first major consideration for correctly identifying indel mutations is the use of assembly based approaches over alignment-based approaches. Assembly based methods afford the best sensitivity for detecting indel mutations, especially long indels, as they avoid any expectation or dependencies of the reads aligning end-to-end to the reference genome. This is especially important for reads sequencing long indels (>30 bp), as most read mapping algorithms typically treat these as soft-clipped reads or fail to map them at all. The next most important consideration is the presence of repeats in the genome, especially near-identical repeats within close proximity to each other and STR sequences that may increase the false-positive rate. Our analysis shows near-identical repeats are widespread in the genome, and if not carefully detected may introduce “false-bubbles” where the reads or assembled contigs incorrectly align to the repetitive sequences. Simple tandem repeats (STRs) are also very challenging, because of their especially high indel error rates and also especially high true mutation rates that can obscure true indels.

Considering the widespread interest to sequence genomes and identify indels for medical and other purposes, it is virtually certain that we will see the rise of new algorithmic and experimental approaches for indel detection in the near future. Algorithmically, we anticipate the development of more specialized methods for detecting indels within complex samples, such as somatic mutations in heterozygous cancer populations. For these samples, new scoring metrics will need to be developed that can accurately recognize low-coverage indels present in only a fraction of the cells. We also anticipate the rise of algorithms that can utilize very large populations of samples, especially to augment the standard reference genome with indels commonly found in the population to improve initial mapping efficiencies and also to flag problematic regions with unusual rates of mutations. Experimentally, we anticipate the rise of new sequencing technologies that can produce longer reads that will improve both micro-assembly and resolving repetitive elements. Already the widely used Illumina chemistry is available to produce ~250 bp reads, or even ~500 bp reads by merging paired-end reads together, and new single molecule approaches (PacBio and Oxford Nanopore) can generate substantially longer reads, although requiring new algorithms to tolerate the high error rate of the technologies. As these and other technologies improve we anticipate our ability to discover indel mutations will improve leading to the discovery of many additional indel-related diseases and phenotypes.

## Conflict of Interest Statement

The authors declare that the research was conducted in the absence of any commercial or financial relationships that could be construed as a potential conflict of interest.

## Footnotes

^1^http://cancergenome.nih.gov/

^2^http://www.broadinstitute.org/gatk/guide/article?id=4146

## References

[B1] Ballantyne K. N. Goedbloed M. Fang R. Schaap O. Lao O. Wollstein A. (2010). Mutability of Y-chromosomal microsatellites: rates, characteristics, molecular bases, and forensic implications. Am. J. Hum. Genet. 87, 341–353. 10.1016/j.ajhg.2010.08.006 20817138 PMC2933352

[B2] Chen K. Chen L. Fan X. Wallis J. Ding L. Weinstock G. (2014). TIGRA: a targeted iterative graph routing assembler for breakpoint assembly. Genome Res. 24, 310–317. 10.1101/gr.162883.113 24307552 PMC3912421

[B3] DePristo M. Banks E. Poplin R. Garimella K. Maguire J. Hartl C. (2011). A framework for variation discovery and genotyping using next-generation DNA sequencing data. Nat. Genet. 43, 491–498. 10.1038/ng.806 21478889 PMC3083463

[B4] Ellegren H. (2004). Microsatellites: simple sequences with complex evolution. Nat. Rev. Genet. 5, 435–445 10.1038/nrg1348 15153996

[B5] Gymrek M. Golan D. Rosset S. Erlich Y. (2012). lobSTR: a short tandem repeat profiler for personal genomes. Genome Res. 22, 1154–1162. 10.1101/gr.135780.111 22522390 PMC3371701

[B6] Highnam G. Franck C. Martin A. Stephens C. Puthige A. Mittelman D. (2013). Accurate human microsatellite genotypes from high-throughput resequencing data using informed error profiles. Nucleic Acids Res. 41, e32. 10.1093/nar/gks981 23090981 PMC3592458

[B7] Iossifov I. O’Roak B. J. Sanders S. J. Ronemus M. Krumm N. Levy D. (2014). The contribution of de novo coding mutations to autism spectrum disorder. Nature 515, 216–221. 10.1038/nature13908 25363768 PMC4313871

[B8] Iqbal Z. Caccamo M. Turner I. Flicek P. McVean G. (2012). De novo assembly and genotyping of variants using colored de Bruijn graphs. Nat. Genet. 44, 226–232. 10.1038/ng.1028 22231483 PMC3272472

[B9] Khurana E. Fu Y. Colonna V. Mu X. J. Kang H. M. Lappalainen T. (2013). Integrative annotation of variants from 1092 humans: application to cancer genomics. Science 342, 1235587. 10.1126/science.1235587 24092746 PMC3947637

[B10] Leggett R. M. Ramirez-Gonzalez R. H. Verweij W. Kawashima C. G. Iqbal Z. Jones J. D. (2013). Identifying and classifying trait linked polymorphisms in non-reference species by walking coloured de Bruijn graphs. PLoS One 8(3):e60058. 10.1371/journal.pone.0060058 23536903 PMC3607606

[B11] Li H. Durbin R. (2009). Fast and accurate short read alignment with Burrows–Wheeler transform. Bioinformatics 25, 1754–1760. 10.1093/bioinformatics/btp324 19451168 PMC2705234

[B12] Li S. Li R. Li H. Lu J. Li Y. Bolund L. (2012). SOAPindel: efficient identification of indels from short paired reads. Genome Res. 23, 195–200. 10.1101/gr.132480.111 22972939 PMC3530679

[B13] Mirkin S. M. (2007). Expandable DNA repeats and human disease. Nature 447, 932–940. 10.1038/nature05977 17581576

[B14] Montgomery S. B. Goode D. L. Kvikstad E. Albers C. A. Zhang Z. D. Mu X. J. (2013). The origin, evolution, and functional impact of short insertion–deletion variants identified in 179 human genomes. Genome Res. 23, 749–761. 10.1101/gr.148718.112 23478400 PMC3638132

[B15] Mose L. E. Wilkerson M. D. Hayes D. N. Perou C. M. Parker J. S. (2014). ABRA: improved coding indel detection via assembly-based realignment. Bioinformatics 30, 2813–2815. 10.1093/bioinformatics/btu376 24907369 PMC4173014

[B16] Narzisi G. O’Rawe J. A. Iossifov I. Fang H. Lee Y. Wang Z. (2014). Accurate de novo and transmitted indel detection in exome-capture data using microassembly. Nat. Methods 11, 1033–1036. 10.1038/nmeth.3069 25128977 PMC4180789

[B17] Pearson C. E. Edamura N. K. Cleary J. D. (2005). Repeat instability: mechanisms of dynamic mutations. Nat. Rev. Genet. 6, 729–742 10.1038/nrg1689 16205713

[B18] Rimmer A. Phan H. Mathieson I. Iqbal Z. Twigg S. R. F. WGS500 Consortium (2014). Integrating mapping-, assembly- and haplotype-based approaches for calling variants in clinical sequencing applications. Nat. Genet. 46, 912–918. 10.1038/ng.3036 25017105 PMC4753679

[B19] Schizophrenia Working Group of the Psychiatric Genomics Consortium. (2014). Biological insights from 108 schizophrenia-associated genetic loci. Nature 511, 421–427. 10.1038/nature13595 25056061 PMC4112379

[B20] The 1000 Genomes Project Consortium. (2010). A map of human genome variation from population-scale sequencing. Nature 467, 1061–1073. 10.1038/nature09534 20981092 PMC3042601

[B21] Treangen T. J. Salzberg S. L. (2011). Repetitive DNA and next-generation sequencing: computational challenges and solutions. Nat. Rev. Genet. 13, 36–46. 10.1038/nrg3117 22124482 PMC3324860

[B22] Weisenfeld N. I. Yin S. Sharpe T. Lau B. Hegarty R. Holmes L. (2014). Comprehensive variation discovery in single human genomes. Nat. Genet. 46, 1350–1355. 10.1038/ng.3121 25326702 PMC4244235

